# Effect of cataract surgery on cognitive function in elderly: Results of Fujiwara-kyo Eye Study

**DOI:** 10.1371/journal.pone.0192677

**Published:** 2018-02-20

**Authors:** Kimie Miyata, Tadanobu Yoshikawa, Masayuki Morikawa, Masashi Mine, Nozomi Okamoto, Norio Kurumatani, Nahoko Ogata

**Affiliations:** 1 Department of Ophthalmology, Nara Medical University, Kashihara, Nara, Japan; 2 Mie Prefectural Mental Care Center, Tsu, Mie, Japan; 3 Department of Community Health and Epidemiology, Nara Medical University, Kashihara, Nara, Japan; Banner Alzheimer's Institute, UNITED STATES

## Abstract

**Purpose:**

To determine whether there is a significant association between prior cataract surgery and cognitive function in an elderly Japanese cohort.

**Setting:**

Nara Medical University, Nara, Japan.

**Design:**

The Fujiwara-kyo Eye Study was a cross-sectional epidemiological study.

**Methods:**

The subjects were ≥ 68-years who lived in the Nara Prefecture and responded to recruitment notices. All of the subjects received comprehensive ophthalmological examinations, and answered questionnaires on their socio-demographic and medical history including prior cataract surgery. The association between prior cataract surgery and cognitive function was determined.

**Results:**

A total of the 2764 subjects whose mean age was 76.3±4.8 years (±standard deviation) was studied. Of these, 668 individuals (24.2%) had undergone cataract surgery. Of these, 150 (5.4%) had dementia as determined by the Mini-Mental State Examination (MMSE) score ≤23, and 877 individuals (31.7%) had mild cognitive impairment (MCI; MMSE score 24–26). The subjects who had prior cataract surgery had significantly lower odds ratio (OR) of having MCI (OR = 0.78, 95% confidence interval; CI 0.64–0.96, *P* = 0.019) than those who had not had cataract surgery after adjusting for age, sex, body mass index, education, hypertension, diabetes, depression, and history of stroke. The OR was still lower when the visual acuity was also added to the adjusted factors (OR 0.79, 95% CI 0.64–0.97, *P* = 0.025). However, prior cataract surgery did not contribute significantly to the low OR for dementia.

**Conclusions:**

Cataract surgery may play a role in reducing the risk of developing MCI independently of visual acuity but not for dementia.

## Introduction

The number of older individuals is rapidly increasing worldwide, and the cost of medical care and social security benefits have correspondingly increased. It is known that both visual and cognitive impairments increase with advancing age [1–3], and these impairments will contribute to the cost of medical care. The incidence of dementia was estimated to be 15% and mild cognitive impairment (MCI) was 13% in individuals ≥65 years in Japan in 2010. Thus, 28% of individuals ≥65 years have some degree of cognitive impairments [4]. Visual and cognitive impairments can limit the activities of daily living and the quality of life (QOL), and earlier studies [5–8] and population-based epidemiological studies [9–14] involving older individuals have shown that there is a strong association between visual impairments and cognitive function. We have also found a significant association between visual impairments and cognitive function in elderly Japanese [3].

Cataract is the most common eye disorder that disturbs the visual acuity in the elderly. Because cataracts are the leading cause of reversible blindness, cataract surgery may be one strategy for decreasing the number of individuals with cognitive impairments.

Thus, purpose of this study was to determine whether there is a significant association between cataract surgery and cognitive function in the elderly.

## Subjects and methods

### The Fujiwara-kyo Study

A detailed description of the Fujiwara-kyo Study has been reported elsewhere [15–18]. Briefly, this was a cohort study whose purpose was to identify factors related to the maintenance of a healthy life, prevention of physical weakness, and improvements of the functional capacities and the QOL of an elderly population in Japan [15–18]. The subjects consisted of residents of the Nara Prefecture who were ≥65-years-old and were living independently in their own homes. The first examinations were performed in 2007 on 4,206 participants. The examinations included a basic interview to obtain socio-demographic data, overall medical conditions, and histories of medical treatments.

### The Fujiwara-kyo Eye Study

An ophthalmological examination was not conducted in the initial survey in 2007. The Fujiwara-kyo Eye study was conducted for the first time as part of the Fujiwara-kyo Study at the second survey between February to November 2012. The data presented in this manuscript were collected from the examinations done in 2012. The subjects recruited at the initial survey in 2007 were ≥ 65-years and were 5-years older in 2012. Eighty new subjects ≥ 65-years were recruited during the survey in 2012.

The surveys were conducted in accordance with the tenets of the Declaration of Helsinki, and the protocol was approved by the Ethics Review Board of Nara Medical University. A signed informed consent form was obtained from all participants. When participants had difficulties reading or understanding the written informed consent form, medical staff members helped them understand the informed consent. If participants could not understand the informed consent, they were not included in this study without obtaining proxy consent from a legally authorized representative. We did not have any participants who assumed and agreed the informed consent but could not conduct MMSE.

### Visual acuity and slit-lamp examinations for cataract

The uncorrected and corrected visual acuities of both eyes were measured with a Landolt ring chart at 5 m. The refractive errors were measured by an autoref/keratometer (ARK-700A, Nidek, Gamagori, Aichi, Japan), and these values were used when the best-corrected visual acuity (BCVA) was measured. The BCVA of the better seeing eye was recorded as the BCVA, and the decimal BCVA was converted to the logarithm of the minimum angle of resolution (logMAR) units for the statistical analyses. The slit-lamp examination was performed by an ophthalmologist to determine the status of the crystalline lens.

### Self-administered cataract questionnaire

A prior cataract surgery was determined by the self-administered questionnaire. The eyes were divided into those with and those without a cataract surgery. The subjects who had undergone cataract surgery in at least one eye were placed in the cataract surgery group for the person wise analyses.

Two hundred and four randomly selected participants were examined by slit-lamp to verify the accuracy of the self-administered questionnaires regarding the cataract surgery. For this, the presence of the crystalline lens or an implanted intraocular lens was determined to confirm that the answer to the self-administered questionnaire was correct. The Kappa coefficient between self-administered questionnaires and diagnosis based on the slit-lamp examination was excellent at 0.95.

### Cognitive and depression examinations

The Mini-Mental State Examination (MMSE) was used as a cognitive function test. This test is commonly used for dementia screening [19], and it consists of 5 downstream items; orientation, memory, attentiveness for calculations, speech function, and design capacity. This test was performed by verbal questioning of 5 to 10 minutes duration by skilled clinical psychologists. The maximum score for the MMSE is 30 points, and individuals with a score of ≤ 23 points were classified as having dementia, those with a score of 24–26 points were classified as having mild cognitive impairments (MCI), and those with a score of ≥27 points were classified as having normal cognitive function [3, 20, 21].

The presence of symptoms of depression was evaluated by a self-administered questionnaire by the Geriatric Depression Scale (GDS-15).

### Socio-demographic data and general information

The educational level, medical history, and information on medications being used were determined from the answers to the self-administered questionnaire. Hypertension was based on a self-reported diagnosis and current antihypertensive therapy. Diabetes mellitus was defined based on self-reported diagnosis, current anti-diabetic therapy, and fasting plasma glucose and glycated hemoglobin levels.

### Statistical analyses

The significance of the differences in the cognitive status by age and BMI were determined by analysis of variance. The differences in the sex and prevalence of hypertension, diabetes, depression, and stroke between the cognitive statuses were determined by chi-square tests. The differences in the BCVA by the cognitive statuses were analyzed by unpaired *t*-tests. Multivariate statistical models were used to analyze the following as covariates; the variables associated with cognitive impairment in Table 1 *(P* <0.25) for model 1 and all variables shown in Table 1 for model 2. Statistical analyses were performed with SPSS (version 22.0; SPSS Inc., Chicago, IL). A *P* <0.05 was taken to be significant.

**Table 1:** Basic and clinical characteristics of 2764 participants by cognitive status.

Characteristics	All	Normal	MCI	Dementia	*P* value
MMSE examination score		≥ 27	26–24	≤ 23	
Number of subjects (%)	2764	1737 (62.8)	877 (31.7)	150 (5.4)	
Basic parameters					
Age, mean ± SD, years	76.3 ± 4.8	75.9 ± 4.6	76.6 ± 5.0	79.2 ± 5.3	<0.001
Sex (male), number (%)	1453 (52.6)	915 (52.7)	449 (51.2)	89 (59.3)	0.18
BMI, mean ± SD, kg/m^2^	22.7 ± 2.9	22.6 ± 2.8	22.9 ± 3.0	22.5 ± 3.0	0.12
Education (≧ 13 years), number (%)	634 (22.9)	454 (26.1)	160 (18.2)	20 (13.3)	<0.001
BCVA, mean ± SD, logMAR units	-0.021 ± 0.13	-0.028 ± 0.13	-0.015 ± 0.13	0.033 ± 0.15	<0.001
Clinical parameters					
Hypertension, number (%)	1370 (52.0)	895 (51.5)	472 (53.9)	70 (46.7)	0.21
Diabetes, number (%)	409 (14.8)	238 (13.7)	146 (16.6)	25 (16.7)	0.11
Depression (GDS ≧6), number (%)	428 (15.5)	259 (14.9)	140 (16.0)	29 (19.3)	0.33
Stroke, number (%)	170 (6.2)	96 (5.5)	63 (7.2)	11 (7.3)	0.21

MCI, mild cognitive impairment; SD, standard deviation; BMI, body mass index; BCVA, best corrected visual acuity; GDS, geriatric depression scale

## Results

Data was available on 2,764 individuals on the status of the crystalline lens or prior cataract surgery (S1 Table). All of these individuals had been examined by MMSE. The mean age of subjects was 76.3 ± 4.8 years (mean ± standard deviation, SD). Of the 2,764 subjects, 5.4% were placed in the dementia group, 31.7% in the MCI group, and 62.8% in the normal cognitive function group. The mean age of the dementia group and the MCI group was significantly higher than that of the normal cognitive function group (*P* <0.001, Table 1). The number of subjects with ≥13 years of education was lower in the dementia group (13.3%) and the MCI group (18.2%) than that in the normal cognitive function group (26.1%; *P* <0.001, Table 1). The mean BCVA was significantly worse in the dementia group and the MCI group than that in the normal cognitive function group (*P* <0.001, Table 1).

### Associations between visual acuity and cognitive impairment by cataract status

There were 24.2% of the subjects in the prior cataract surgery group. Dementia was present in 4.7% and MCI in 32.4% in this group. Dementia was present in 7.6% and MCI was present in 29.5% in the no cataract surgery group. The mean BCVA was significantly better in the normal cognitive function group than in the MCI and the dementia groups in both the prior cataract surgery group (*P* for trend = 0.01) and no cataract surgery group (*P* for trend < 0.01, Table 2).

**Table 2:** Associations between visual acuity and cognitive impairment by cataract status.

	All	Normal	MCI	Dementia	
	(n = 2764)	(n = 1737)	(n = 877)	(n = 150)	*P* for trend
Cataract surgery, number (%)	668 (24.2)	420 (63.8)	197 (32.4)	51 (4.7)	
BCVA, mean ± SD, logMAR units	-0.029 ± 0.14	-0.037 ± 0.14	-0.021 ± 0.14	0.013 ± 0.16	0.01
No cataract surgery, number (%)	2096 (75.8)	1317 (62.9)	680 (29.5)	99 (7.6)	
BCVA, mean ± SD, logMAR units	-0.018 ± 0.13	-0.025 ± 0.13	-0.013 ± 0.13	0.043 ± 0.15	0.01

MCI, mild cognitive impairment; BCVA, best-corrected visual acuity; SD, standard deviation

### Association between cataract surgery and cognitive status

In the multivariate logistic regression analyses, the odds ratio (OR) for MCI was significantly lower in the prior cataract surgery group than in the no cataract surgery group (OR 0.78; *P* = 0.019, model 1, Table 3 and S2 Table). In addition, when the BCVA was included as a variant, the OR for MCI was still significantly lower in the cataract surgery group than that in the no cataract surgery group (OR, 0.79; *P* = 0.025, model 2, Table 3 and S2 Table).

**Table 3:** Odds ratio for association between cataract surgery and cognitive status.

	0R for MCI			0R for Dementia		
Cataract surgery	Age-adjusted	Model 1	Model 2	Age-adjusted	Model 1	Model 2
No cataract surgery	1.00 (ref)	1.00 (ref)	1.00 (ref)	1.00 (ref)	1.00 (ref)	1.00 (ref)
Cataract surgery	0.81	0.78	0.79	1.09	1.10	1.20
(CI)	(0.66–0.99)	(0.64–0.96)	(0.64–0.97)	(0.75–1.60)	(0.75–1.62)	(0.81–1.77)
p value	0.036	0.019	0.025	0.65	0.64	0.36

OR, odds ratio; MCI, mild cognitive impairment; CI, confidence interval; BCVA, best corrected visual acuity

Model 1; Adjusted for all variables shown in Table 1 except BCVA

Model 2; Adjusted for all variables shown in Table 1 including BCVA

In the multivariate logistic regression models, the OR for dementia was not significantly different between the cataract surgery group and the no cataract surgery group after adjusting for confounding factors except for the BCVA (OR 1.10; *P* = 0.64, model 1, Table 3 and S2 Table). When the BCVA was included as a variant, the OR for dementia was still not significant (model 2, Table 3 and S2 Table).

## Discussion

Our results showed that prior cataract surgery significantly reduced the risk of having MCI (OR = 0.78) but did not reduce the risk for dementia. Cataracts are frequent in the elderly and are the leading cause of visual impairments in the elderly worldwide. An earlier study reported that there was a significant correlation between the visual acuity and cognition [22], and subjects with good visual acuity had a 63% lower risk for developing dementia over an 8.5-year period [23]. Similarly, elderly individuals with decreased visual acuity were 5 times more likely to show diminished cognitive performance than elderly individuals with good vision [23]. We also reported earlier that the prevalence of both cognitive and visual impairments increased with increasing age in this cohort [3]. Furthermore, subjects with mild visual impairments (BCVA >0.2 logMAR unit) had 2.4 times higher risk of having dementia than those without visual impairments after adjusting for age, sex, and length of education. These results indicate that it is important to maintain good visual acuity to reduce the risk of developing dementia, however the effects of cataract surgery on preventing cognitive impairment were not analyzed in the Fujiwara-kyo study.

For patients whose poor vision can be attributed to cataracts, surgical intervention may not only improve their vision but also improve their mental status [24–30]. Ishi et al. reported that cataract surgery significantly improved the vision-related QOL in elderly patients, and cognitive impairments and depressive mental status also improved in parallel with the improvement in the vision-related QOL [29]. However, Elliott et al [31] found that vision improvement after cataract surgery or refractive correction (eye glasses) did not improve the short-term cognitive function.

A report from UK [32] emphasized the use of cataract surgery in those with both normal and impaired cognition, and it was reported that both groups had significant improvements in the visual outcomes. Cognitive impairment may, however, limit the visual improvements following cataract surgery. We also found that the mean BCVA was significantly better in the normal cognitive function group than that in the cognitive impaired group in both the prior cataract surgery and no cataract surgery groups. Thus, patients with impaired cognition benefit from cataract surgery but not to the same extent as patients with normal cognition.

We evaluated both dementia and MCI and found that 37.2% of the subjects had MCI. This was comparable to the prevalence of MCI that was reported to be 42% in an older population in the United Kingdom [33].

The results of an earlier study showed that cognitive and visual function improvements could not be attributed to cataract surgery per se, and the investigators concluded that cataract surgery does not affect cognitive function [34]. However, our results demonstrated that individuals who had cataract surgery were less likely to have MCI but not dementia, and it was independent of the improved visual acuity. This is probably because MCI is a preventable and reversible condition [35, 36].

We showed earlier in the HEIJO-KYO Cohort that cataract surgery with the implantation of an IOL was significantly associated with a lower incidence of MCI in multivariate statistical models adjusting for visual BCVA and several major causes of cognitive impairments [21]. In the HEIJO-KYO Cohort, we did not have sufficient number of participants with dementia who had MMSE score of ≤23. Thus, in the current study, we divided cognitive impairment subjects into those with dementia and those with mild cognitive impairment and found the differences.

Cataract surgery markedly increases the amount of light reaching the retina. This is important because light is a primary environmental cue for regulating the biological clock, and a desynchronization of the circadian rhythm can predispose the individual to cognitive impairments [37, 38]. Ayaki et al. reported that cataract surgery improved the sleep quality by increasing light transmittance needed to maintain the circadian rhythm [39]. Thus, we suggest that the increase in light impinging on the retina might be one mechanism for preventing cognitive impairments.

There are some limitations in this study. First, this was a cross-sectional study which precludes assessments of causality although earlier pretest–posttest studies supported the beneficial effects of cataract surgery on cognitive functions. Thus, further longitudinal studies of longer durations are needed. Second, we used MMSE to determine the dementia and MCI. To diagnose dementia and MCI accurately, MMSE may not be enough. However this was a survey study, and extensive examinations of cognitive function were limited.

In conclusion, cataract surgery may be important in reducing the risk of developing MCI but not for dementia independently of the improvements of the visual acuity.

## Supporting information

S1 Table Minimal dataset for basic and clinical characteristics. (XLSX)

S2 Table Odds ratio for the association between cataract surgery and cognitive status. (XLSX)
